# Prognostic Factors for Responders of Home-Based Pulmonary Rehabilitation—Secondary Analysis of a Randomized Controlled Trial

**DOI:** 10.3390/healthcare13030308

**Published:** 2025-02-03

**Authors:** Chul Kim, Hee-Eun Choi, Chin Kook Rhee, Jae Ha Lee, Ju Hyun Oh, Jun Hyeong Song

**Affiliations:** 1 Department of Rehabilitation Medicine, Sanggye Paik Hospital, Inje University College of Medicine, Seoul 01757, Republic of Korea; josephck@naver.com; 2 Department of Physical Medicine and Rehabilitation, Haeundae Paik Hospital, Inje University College of Medicine, Busan 48108, Republic of Korea; snsmd7@gmail.com; 3 Share and Service Inc., Busan 48002, Republic of Korea; 4 Division of Pulmonary and Critical Care Medicine, Department of Internal Medicine, Seoul St. Mary’s Hospital, College of Medicine, The Catholic University of Korea, Seoul 02812, Republic of Korea; chinkook77@gmail.com; 5 Division of Pulmonology and Critical Care Medicine, Department of Internal Medicine, Haeundae Paik Hospital, Inje University College of Medicine, Busan 48108, Republic of Korea; anilleus@naver.com; 6 Department of Pulmonary and Critical Care Medicine, Sanggye Paik Hospital, Inje University College of Medicine, Seoul 01757, Republic of Korea; s5667@paik.ac.kr

**Keywords:** rehabilitation, digital health, secondary prevention, prognosis, chronic respiratory disease

## Abstract

Background: Home-based pulmonary rehabilitation (PR) is an effective alternative to center-based PR. However, not all participants exhibit sufficient therapeutic improvement, highlighting the need to identify appropriate candidates to maximize cost-effectiveness. This study aimed to identify the factors associated with favorable outcomes in home-based PR, focusing on the role of digital therapeutics (DTx). Methods: This secondary analysis used data from a randomized controlled trial. Participants with chronic respiratory disease (CRD) were divided into responders and non-responders based on a change in 6 min walk distance (6MWD) and patient-reported outcome measures (PROM) representing dyspnea and health-related quality of life. Factors such as baseline 6MWD, DTx use, and pulmonary function were analyzed for their predictive value in improving 6MWD and PROM scores. Results: Responders, particularly those using DTx, showed significantly greater improvements in 6MWD than non-responders. Participants with a baseline 6MWD under 500 m demonstrated a higher likelihood of exceeding the minimum clinically important difference in 6MWD. DTx use strongly predicted improvements in both 6MWD and PROM scores. The baseline diffusing capacity of the lungs for carbon monoxide was also a significant factor influencing improvements in the modified Medical Research Council scale. Conclusions: Responders to 8-week program of home-based PR exhibited a relatively lower baseline health status. Encouraging participants with poorer baseline health could improve adherence to PR and enhance cost-effectiveness. Additionally, improvements in 6MWD and PROM scores were associated with the use of DTx. Considering the functions of DTx, proper supervision for home-based exercise may be crucial for achieving optimal outcomes.

## 1. Introduction

Traditionally, pulmonary rehabilitation (PR) has been viewed as a “last ditch” effort for patients with chronic respiratory disease (CRD), and most PR programs enrolled individuals with severe respiratory impairment [1]. As evidence supporting the effectiveness of PR has been established, it is now recognized as a core component of treatment in patients with CRD and is recommended for patients in less severe stages [2]. Despite its proven benefits, PR remains underutilized globally, with less than 5% of eligible patients with chronic obstructive pulmonary disease (COPD) receiving it, even though COPD comprises the largest proportion of PR referrals [1,2].

The reasons for the poor attendance and high dropout rates in PR are multifactorial [3]. Barriers to PR may include limited numbers and availability of PR programs, with an insufficient number of related healthcare professionals. Suboptimal referrals of patients from other healthcare professionals who are unaware of the process and benefits of PR may also play a role. Additionally, patient-related barriers to attendance, such as issues associated with travel and transportation or racial and socioeconomic disparities, also exist [2]. Poor transport infrastructure and lack of insurance coverage in developing countries may exacerbate these barriers [2,3].

Typically, center-based PR, in which direct supervision is provided at the PR center, is the conventional form for delivering PR. However, to overcome the above barriers, home-based PR models have emerged as an alternative to conventional center-based PR, which delivers essential components of PR in home settings [4]. Recent evidence suggests that home-based PR is as effective as center-based PR [2,4]. The safety of home-based PR has also been confirmed, even in individuals with severe COPD, using long-term supplemental oxygen [2].

However, home-based self-exercise has some pitfalls without monitoring devices and supervision. The target heart rate is important for determining the appropriate exercise intensity and establishing a safety zone for home-based self-exercise [5]. Commonly, patients are instructed to take pulses at their radial or carotid arteries during exercise; however, this method is difficult, inconvenient, and may be inaccurate [5]. Additionally, the target heart rate, often estimated based on age, tends to differ significantly from the actual target heart rate values derived from cardiopulmonary exercise tests [6]. Unsupervised exercise in participants undergoing inappropriate or inadequate exercise may account for suboptimal therapeutic effects in some home-based rehabilitation programs [7].

With recent technological developments, commercial electronic devices with heart rate monitors have been introduced, and their excellent validity and accuracy have been verified [5]. The emerging concept of digital therapeutics (DTx) extends beyond the scope of mere measurement tools. DTx refers to software and medical devices that provide evidence-based therapeutic interventions to prevent, manage, and treat diseases [8]. DTx can be synchronous (live video conferencing and remote patient monitoring), asynchronous, or in the form of mobile health [9]. The coronavirus disease 2019 (COVID-19) pandemic has also necessitated the remote delivery of treatment for social distancing and infection control [3,7], thereby accelerating the development and related research of DTx.

The authors previously developed and validated the EASYBREATH application (app), a PR program comprising aerobic exercise training, breathing retraining, and educational programs [8]. Home-based PR guided by the EASYBREATH app in patients with CRD significantly improved exercise capacity, alleviated dyspnea, and enhanced overall quality of life at 8 weeks [8]. The therapeutic efficacy of the EASYBREATH application has resulted in it being designated as South Korea’s fourth DTx device by the Ministry of Food and Drug Safety.

However, recent work has shown that there are insufficient resources to provide center-based PR to all patients with candidate respiratory disease, and optimizing limited healthcare resources through patient selection is crucial [10]. Previous studies have reported that more than one-third of participants did not respond adequately to center-based PR, even after completing the program [10,11,12]. Therefore, efforts have been made to identify the clinical and functional predictors of success or failure after center-based PR [10,11,12,13,14].

In the present study, we aimed to identify the determinants associated with favorable outcomes after home-based PR using data from an original randomized controlled trial (RCT) [8]. For subsequent analysis of previous research on home-based PR [8], the authors also conducted a secondary analysis of data from the original RCT to identify predictors of success in terms of improving exercise capacity, health-related quality of life (HRQOL), and alleviating dyspnea, especially regarding the utilization of DTx. To the best of our knowledge, this is the first study to determine factors that could predict responders and non-responders among home-based PR participants.

## 2. Materials and Methods

This was a secondary data analysis from a multi-center, prospective, RCT that investigated the efficacy and safety of the DTx application for PR and EASYBREATH. The main outcomes of the original study have been previously published [8].

The research protocol to evaluate the efficacy and safety of EASYBREATH 1.0.0 was approved by the Korean Ministry of Food and Drug Safety (Approval No. 1431). This study was approved by the Institutional Review Board of the Catholic University Seoul St. Mary’s Hospital (Approval No. KC23DNDS0031), the Inje University Haeundae Paik Hospital (Approval No. 2022-12-013-002), and the Inje University Sanggye Paik Hospital (Approval No.2022-11-022-001). All the participants provided written informed consent.

### 2.1. Overview of the Randomized Controlled Trial and Main Findings

The main study protocol involved patients with CRD undergoing home-based PR with (DTx group) or without (control group) digital guidance for 8 weeks. The participants were randomly assigned to either group and assessed at baseline and 4 and 8 weeks post-intervention [8].

The sample size required for this clinical trial was calculated as follows: Based on the study by Pradella et al. [15], the mean changes in the 6 min walk distance (6MWD) after 8 weeks of post-treatment compared to baseline were assumed to be 5.5 m and 65.7 m for the control and experimental groups, respectively, with a standard deviation of 92.9 m. A two-sample *t*-test with equal variance was applied under a two-tailed significance level of 0.05 and a statistical power of 80%. Using PASS version 21.0.9 (NCSS, LLC, Kaysville, UT, USA) for sample size calculation with a 1:1 randomization ratio, the required sample size was determined to be 39 participants per group. Assuming a 15% dropout rate, a total of 92 participants (46 per group) were enrolled in this trial. Randomization was performed using a random number generator computerized with a block randomization method in SAS version 9.4 (SAS Institute, Inc., Cary, NC, USA), without stratifying participants based on their baseline characteristics [8].

The inclusion criteria of the original RCT [8] were as follows: (1) Diagnosis with COPD, lung cancer, asthma, bronchiectasis, etc., and requiring PR due to respiratory symptoms such as dyspnea or difficulties in daily life. (2) Obstructive (ratio of forced expiratory volume in one second to forced vital capacity (FEV1/FVC) < 0.7) or restrictive (predicted FVC < 0.8) abnormalities in pulmonary function tests. (3) No difficulty using mobile applications on equipment such as smartphones or tablet computers and age ≥19 years. The exclusion criteria were poor cognition, previous participation in PR, unstable cardiovascular disease, walking difficulties, and pregnancy.

The CG participated in a single supervised exercise session and was instructed to exercise independently at least three times weekly for 8 weeks. The DTx group was supported by the EASYBREATH app, which includes an initial and repeat 6 min walk test (6MWT), an aerobic exercise training program, breathing retraining, and a PR educational program [8]. Following the initial 6MWT, an individualized exercise prescription was developed for each patient using the app’s unique exercise prescription algorithm. Subsequently, aerobic exercise training was carried out according to the individualized exercise prescriptions. An exercise report was provided and patients provided feedback on exercise intensity at the completion of each session. If a patient perceived the intensity to be too low, a repeat 6MWT was conducted, and a new exercise prescription was adjusted accordingly. Breathing retraining and PR education were conducted through video sessions.

The outcome measures included the 6 min walk distance (6MWD) and patient-reported outcome measures (PROM) scores, specifically the modified Medical Research Council (mMRC) dyspnea scale, COPD assessment test (CAT), St. George’s Respiratory Questionnaire (SGRQ) total score, and the Hospital Anxiety and Depression Scale (HADS).

In brief, the change in 6MWD after 8 weeks, which was the primary outcome of the original study, demonstrated a significant difference between the DTx and control group (57.68 m vs. 21.71 m). The change in mMRC scores (−0.33 vs. 0.10), CAT scores (−4.86 vs. 1.29), and total SGRQ scores (−3.32 vs. 3.11) also showed a significant difference between the groups after 8 weeks [8].

### 2.2. Analysis of 6 Min Walk Distance Results

Response to home-based PR was defined as achieving a minimum clinically important difference (MCID) for each parameter, and the authors aimed to identify factors that predict surpassing the MCIDs for each parameter. For the primary outcome, improvement exceeding the MCID in 6MWD (54 m) was demonstrated only in the DTx group. The baseline characteristics and physiologic tests of responders and non-responders in 6MWD were compared. Additionally, the 6 min walk test is a simple, physiologic test that offers an instant, rough evaluation of a patient’s general cardiorespiratory fitness (CRF) prior to lobectomy. A previous study revealed that patients who walked <500 m within 6 min showed significantly higher complication rates after lung resection [16,17]. The authors postulated that participants with relatively poor baseline CRF would present different baseline statuses and respond differently to PR than their counterparts.

Since not all participants demonstrated adequate improvement in 6MWD after completing the PR sessions [11], the change in the 6 min walk test from baseline was analyzed by categorizing participants into the following groups: “Exceed MCID”, “Gradual improvement”, “Gradual improvement over MCID”, “Gradual decline”, and “Deterioration from baseline”, thereby reflecting the diversity of PR responses. Using this categorization, participants were divided based on “the use of DTx devices” and “whether the initial 6MWD exceeded 500 m”, and the changes in the 6MWD were analyzed accordingly.

All data were analyzed using SPSS Statistics, Version 25.0 (IBM Corp.), and values are presented as mean and standard deviation (M ± SD) values or as numbers and percentages. The *t*-test was used to compare the mean values between the groups. Baseline characteristics showing a significant difference between responders and non-responders (*p* < 0.05) in terms of each outcome parameter were entered into the regression analysis. Univariate and multivariate logistic regression analyses were performed to evaluate the presence of independent predictors of the efficacy of home-based PR, which was expressed as a significant increase in the 6MWD exceeding the MCID. These were expressed as binary dependent variables and included alternatively in the logistic regression (0, no improvement exceeding the MCID; 1, improvement exceeding the MCID).

### 2.3. Analysis of Patient-Reported Outcome Measures

Since the HADS failed to show a significant difference between the two groups, the subsequent analysis of the patient-reported outcome measures (PROM) scores included the mMRC, CAT, and SGRQ total scores. In the case of the CAT and SGRQ total scores, a decrease of 2 and 4 points, respectively, which are their known MCIDs, was considered indicative of a response to PR [18,19].

However, the mMRC scoring system is structured in units of 0 to 4, and the average improvement MCID for the group has been reported as 0.5 [20]. Therefore, if an individual’s mMRC score decreased by one, they were considered to have responded to PR.

Logistic regression analysis was performed to identify the factors associated with exceeding the MCID of each PROM score, as it was for the 6MWD. Additionally, to analyze whether changes in the primary outcome measure led to changes in the secondary outcome measure, the correlations among the changes in outcome parameters were examined using a simple correlation analysis. Variables showing a significant correlation (*p* < 0.05) were entered into a simple linear regression analysis to arithmetically describe the relationship between changes in the two variables.

## 3. Results

For baseline characteristics, all variables, except for the allocated groups, showed no significant difference between responders and non-responders in the 6MWD (Table 1). Among the 6MWD responders, 67.7% utilized DTx during home-based PR, whereas 60.9% of the non-responders engaged in self-directed exercise at home (*p* < 0.05). Regarding DTx usage, 53.8% of the DTx group achieved an improvement exceeding the MCID in 6MWD, compared to only 26.3% of the CG. The baseline primary and secondary outcome parameters showed no significant differences between groups.

Participants were also divided into two groups based on the baseline 6MWD with a threshold of 500 m (Table 2). Participants in the DTx group and the CG were evenly distributed when stratified by an initial 6 min walk distance of approximately 500 m. Participants exceeding 500 m initially showed higher oxygen saturation during rest, with a higher percentage of predicted values in forced expiratory volume in one second (FEV1) and FEV1 over forced vital capacity (FVC), better HRQOL represented by baseline SGRQ total score, and better 6MWD results. This tendency persisted after PR, and the “exceeding group” constantly showed better 6MWD and SGRQ total score results. mMRC, which showed no significant difference at baseline, also showed a significant difference with improvement only shown in the “exceeding group”.

Both the use of DTx during home-based PR and whether the participants exceeded the 500 m threshold in the baseline 6MWD were considered. These factors were incorporated into the analysis based on changes in the 6MWD from baseline to week 8 (Table 3). The proportion of participants showing a tendency towards improvement in the 6MWD was significantly greater in the DTx group (*p* < 0.05). In addition, participants who did not exceed 500 m in the baseline 6MWD demonstrated a significantly higher proportion of gradual improvement, surpassing the MCID in the 6MWD (*p* < 0.05). In logistic regression analysis, considering “gradual improvement surpassing MCID in 6MWD” as the dependent variable, an initial 6MWD of less than 500 m was identified as a significant predictor (*p* = 0.04, Exp(B) = 0.33, 95% CI = 0.11–0.96; not presented in the table).

Prior to conducting a regression analysis of the outcome measures, a *t*-test was performed to identify potential predictors by comparing groups based on whether the outcome measures were achieved. Following a series of *t*-tests, seven potential independent variables (allocated groups, age, sex, diffusing capacity of carbon monoxide (DLCO), current smoking, comorbid liver disease, and cancer) were identified and subsequently included in multivariate logistic regression analysis (Table 4). The utilization of DTx was revealed to be a predictor of exceeding the MCID in PROM scores. In the case of the mMRC, baseline DLCO was also identified as a factor influencing improvement.

Additionally, when considering the achievement of the MCID in the 6MWD as the dependent variable, the use of DTx was confirmed to act as a predictor of improvement, consistent with other outcome parameters (*p* = 0.01, Exp(B) = 3.27, 95% CI = 1.25–8.51; not presented in the table). In a supplementary logistic regression analysis, where the dependent variable was defined as “individuals experiencing deterioration compared to baseline”, DLCO emerged as a significant predictor of deterioration (*p* = 0.03, Exp(B) = 1.17, 95%CI = 1.02–1.35; not presented in the table).

**Figure 1:**
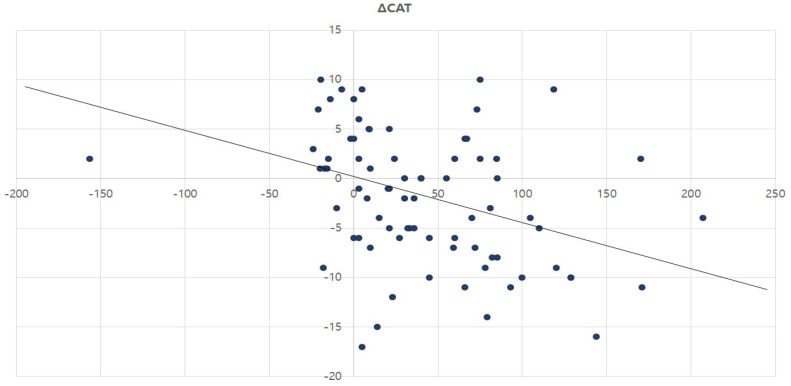
Relationship between changes in 6MWD and changes in CAT score.

Furthermore, Pearson’s correlation analysis was conducted to examine whether the increase in the primary outcome measure, 6MWD, led to a significant improvement in HRQOL and dyspnea. The change in 6MWD at week 8 was negatively correlated with both the CAT score at week 8 and the change in the CAT score during the same period. In contrast, the SGRQ showed no correlation with the change in the 6MWD (Table 5). Additional simple regression analysis demonstrated a weak negative association between the change in 6MWD and the change in the CAT score (β = −0.05, *p* = 0.002). The model accounted for 13% of the variance in the changes in quality-of-life scores (R^2^ = 0.13) (Figure 1).

## 4. Discussion

Previous studies have attempted to identify factors that can predict which patients are likely to derive therapeutic benefits from center-based PR [10,11,12,13,14]. It has been reported that gains can be achieved from center-based PR regardless of age, sex, lung function, or smoking status [1]. In more recent studies, factors such as age, body mass index, dry lean mass, and physiological parameters, including resting PaO2, hemoglobin levels, erythrocyte count, hematocrit, and iron levels, have been identified as predictors of favorable outcomes in center-based PR [10,11,12]. In the present study, we aimed to identify the predictors of favorable outcomes after home-based PR.

### 4.1. Changes in Outcome Parameters Following Home-Based Pulmonary Rehabilitation

6MWD, the primary outcome measure of the original study, is one of the most widely accepted outcome measures in PR studies. It is also a well-recognized outcome measure in other chronic conditions such as pulmonary hypertension and chronic heart failure [21]. The MCID of the 6MWD varies across the literature, ranging from as little as 25 m to as much as 54 m [1]. Regardless of the selected value, the CG demonstrated an average change in 6MWD of 21 m, which failed to reach the MCID for 6MWD [8]. PR responders, defined as participants achieving an improvement of >54 m in the 6MWD, included a significantly larger proportion of participants using DTx for home-based exercise (Table 1), with a threefold increase in the probability of exceeding the MCID at week 8.

The 6 min walk test has been used to monitor treatment response and establish prognosis in patients with cardiopulmonary diseases [16,17]. Preoperative 6MWD has also been used to predict postoperative mortality following lung resection, with threshold values ranging from 300 to 500 m [16,17]. In the present study, we divided the participants based on a threshold of 500 m in the initial 6MWD. Patients with an initial 6MWD exceeding 500 m had better results in airway obstruction-related parameters on pulmonary function tests, as well as better SGRQ scores, reflecting better health status in terms of lung physiology and HRQOL (Table 2). However, there were no significant differences in other PROM scores, such as the mMRC and CAT scores. This may be because the SGRQ evaluates a broader range of domains and has a more detailed scoring system, allowing it to reflect participants’ QoL more sensitively than the mMRC and CAT [22].

It has been reported that 33–55% of individuals participating in PR show minimal or no improvement in the 6MWD [11]. The change in 6MWD during 8 weeks in the participants was classified into five categories: three patterns of improvement and two patterns of decline (Table 3). The DTx group included a significantly larger proportion of participants with improvement in the 6MWD. Although not statistically significant, fewer DTx users exhibited a pattern of decline compared with the CG (*p*-values of 0.08 and 0.09), suggesting that statistical significance might be achieved with a larger sample size (Table 3).

**Figure 2:**
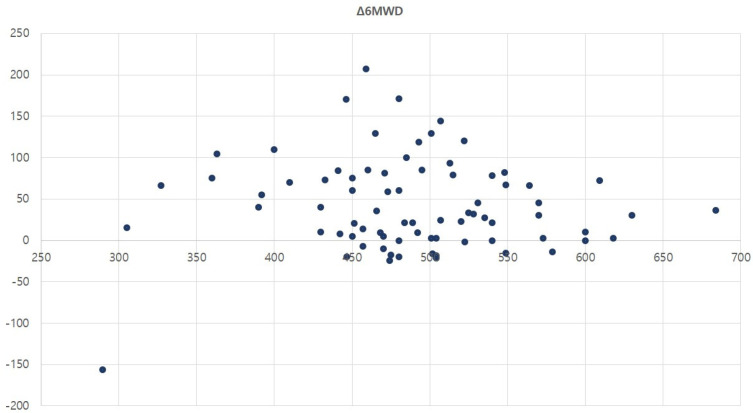
Scatter plot of baseline 6MWD and change in 6MWD after pulmonary rehabilitation.

The group with an initial 6MWD below 500 m showed a significantly higher proportion of participants who demonstrated gradual improvement and surpassed the MCID than the group with 6MWD above 500 m. In the logistic regression analysis, it was found that when the initial 6MWD was greater than 500 m, the probability of demonstrating a “gradual increase surpassing MCID” is reduced to one-third. In addition, the number of participants who “exceeded the MCID” and those showing “gradual decline” was also higher in the group with an initial 6MWD below 500 m. Both trends had a *p*-value of 0.08, suggesting that the differences could become statistically significant with a larger sample size. A scatter plot of the initial 6MWD on the X-axis and the change in the 6MWD on the Y-axis showed that significant improvements were observed around the initial value of 450 m (Figure 2). However, as the initial value decreases, the magnitude of improvement tends to decrease. Combining the above findings, it can be interpreted that an initial 6MWD below 500 m is associated with a greater likelihood of recovery. However, if the initial 6MWD is excessively low, the magnitude of improvement may be reduced.

The findings of this study align with those of previous research in that individuals with lower baseline exercise capacity tend to show greater improvement [11,23]. This pattern can also be observed in cardiac rehabilitation, which shares key components and mechanisms of treatment with PR, in which participants with the lowest initial fitness levels tend to show the most significant improvements [7]. Individuals with greater initial walking capacity may experience a smaller margin for further improvement during PR because of potential physiological or biomechanical “ceiling effects”, as previously reported [11]. In addition to lower baseline exercise capacity, lower health status and lung function have been associated with greater improvement [10,11]. However, it is interesting to note that participants with very severe dyspnea at baseline, classified as mMRC grade 5, demonstrated smaller improvements in the 6MWD than those with less severe dyspnea, as observed in the present study, where participants with excessively low initial 6MWD also showed less improvement [10].

For mMRC, baseline DLCO in absolute values was also identified as a factor influencing improvement (Table 4). Specifically, a 1-point increase in the absolute value of DLCO was associated with a twofold reduction in the probability of improvement in mMRC. Additionally, DLCO was a significant independent predictor of deterioration from baseline, with each 1-point increase in the absolute DLCO value increasing the probability by 1.17-fold. DLCO serves as an indicator of disease severity and treatment response in COPD and is a predictor of all-cause mortality and respiratory complications before lung resection [24,25,26]. Regarding predictors of response to PR, one study reported that participants with severe diffusion defects, indicated by low DLCO, showed greater improvement in the mMRC dyspnea scale, which is consistent with the present study’s findings [23].

We analyzed the impact of improvement in exercise capacity, as represented by the 6MWD, on the actual improvement in HRQOL and dyspnea (Table 5). Changes in the 6MWD showed weak and moderate negative associations with changes in the CAT and CAT scores at week 8, respectively. Linear regression analysis confirmed that the change in 6MWD significantly predicted the change in the CAT score. However, with an explanatory power of only 13.2%, its predictive ability was limited (Figure 1). Although the 6MWD is a good objective measure for PR programs, it is unclear how it translates into improvements in daily life activities [1]. Discrepancies have been reported between what the patient is able to do and what the patient subjectively believes they can do, which aligns with the findings of the present study [21]. A comprehensive evaluation of the efficacy of PR in individuals with COPD is complex; therefore, a combination of assessment tools may be used to obtain a detailed understanding of the patient’s condition [27,28,29].

### 4.2. Digital Therapeutics and Pulmonary Rehabilitation

The use of DTx was associated with favorable outcomes in HRQOL and dyspnea-related measures, with the probability of improvement increasing by 22.7-fold for mMRC, 11.1-fold for CAT, and 9.4-fold for the SGRQ total score (Table 4). To date, home-based PR programs have been demonstrated as effective alternatives to conventional center-based PR, where face-to-face supervision is available [30,31,32]. However, the medical literature lacks consensus owing to variations in study designs and a lack of standardization among procedures. Furthermore, since most prior studies have been conducted under remote supervision, such as home visits or phone consultations with physiotherapists or doctors, there are limited data available on truly “unsupervised” rehabilitation programs [30].

In the present study, the CG participated in a single supervised exercise session and was instructed to exercise independently at least three times weekly for 8 weeks, thereby meeting the true definition of “unsupervised” rehabilitation [8]. On the contrary for DTX group, the medical staff monitored participation in PR supported by EASYBREATH app and reviewed exercise data on the medical staff web page. Additionally, patients were contacted via phone if they did not meet the predefined minimal adherence criteria (failure to participate in exercise three consecutive times) [8]. DTx app usage was categorized based on the percentage of adherence to pre-designated exercise schedules as follows: Very High (≥80%), High (>60% to <80%), Moderate (>40% to ≤60%), Low (>20% to ≤40%), and Very Low (<20%). Notably, every subject in the DTx group showed very high compliance with the app, participating in >80% of the predesignated sessions [8]. It would have been valuable for the authors to assess exercise adherence, including frequency and intensity, in the CG. Regrettably, such data were not obtained for the CG [8].

In a previous study comparing the effects of center- and home-based cardiac rehabilitation (HBCR) during the COVID-19 pandemic, it was found that unsupervised HBCR resulted in significantly less improvement than previously reported, likely due to a lack of adequate supervision [7]. The authors believe that this difference may also explain the suboptimal improvement observed in the 6MWD of the CG, which increased by only 21 m, less than the 50 m typically recognized as the average improvement associated with PR [1,8].

Digital health refers to diverse technologies and strategies aimed at delivering virtual medical, health, and educational services. Rather than representing a specific intervention, it serves as a means to enhance care delivery and education [9]. On the other hand, DTx is considered as a subset of digital medicine, which falls under the broader category of digital health. Digital health, digital medicine, and DTx products vary in clinical evidence requirements and regulatory oversight due to differing claim and risk levels [33]. However, no universally accepted definition of digital therapeutics (DTx) exists, leading to varying interpretations and applications across countries and research institutions. Republic of Korea is the only country to define DTx at the government level as “software classified as a medical device that delivers evidence-based therapeutic interventions to prevent, manage, or treat medical disorders or diseases” [33]. The Digital Therapeutics Alliance, a leading organization in defining and promoting DTx, defined it as “evidence-based therapeutic interventions delivered via high-quality software programs to treat, manage, or prevent diseases or disorders” [33].

With the advancement of technology and emergence of the COVID-19 pandemic, social distancing measures have highlighted the importance of tele-rehabilitation, in which the core components of rehabilitation are delivered remotely. This is especially important in PR because many patients have limited access to PR programs due to distance. In Korea, approximately 70% of participants undergoing PR are treated at tertiary hospitals, which are predominantly located in major cities or provinces such as Seoul, Gyeonggi, Busan, and Jeonbuk [34,35]. Residing in rural areas or a lack of local provision of PR services poses a barrier to accessing PR in other countries [36,37]. Although home-based PR has emerged as a potential solution to overcome these barriers, its effectiveness appears to be significantly diminished in the absence of appropriate guidance and supervision. This is likely to be even more pronounced in countries where reimbursements for home visits or remote consultations have not been established. In this context, DTx holds promise in bridging this gap by remotely guiding patients through exercise regimens and providing app-based feedback tailored to their activity levels.

### 4.3. Limitations of the Present Study

This study had a few limitations. First, as with all retrospective analyses, the validity of the data is inherently constrained by the study design, which yields a lower level of evidence than prospective studies. Second, the sample size was relatively small despite the original multi-center study design. Another limitation is the lack of assessment of exercise adherence (e.g., frequency, intensity, duration) in the CG. While the exercise supervision of the DTx group was quantified via digital devices, no objective measurements were available for comparison in the CG, thereby weakening the strength of the conclusions. Fourth, the results of the analyses lacked coherence and were sparse, making it difficult to draw unified conclusions. However, the statistical methods employed in this study were appropriately selected and applied in accordance with context, which is considered a strength of the methodology. Additionally, the prognostic variables identified in previous studies were not significant. This may be attributable to the small sample size, which may have yielded statistically significant results in a larger cohort.

## 5. Conclusions

Responders to home-based PR in terms of 6MWD exhibited a lower baseline health status than non-responders. Encouraging participants with poorer baseline health could not only improve adherence to PR but also enhance cost-effectiveness. Additionally, improvements in exercise capacity, HRQOL, and the alleviation of dyspnea were associated with the use of DTx. Considering the functions and roles of DTx, proper supervision to ensure that patients performing self-exercise at home maintain adequate exercise intensity may be crucial for achieving optimal outcomes.

## Figures and Tables

**Table 1:** Baseline characteristics of responders and non-responders in 6 min walk test after 8 weeks of home-based PR.

Characteristics		Responders (n = 31)	Non-Responders (n = 46)	*p*-Value
Group (DTx/CG ratio)		21:10	18:28	0.01
Age (years)		66.7 ± 7.1	64.8 ± 10.1	0.36
Sex, male/female ratio		25:6	39:7	0.64
Height (m)		1.64 ± 0.7	1.67 ± 0.6	0.05
Weight (kg)		64.3 ± 14.9	64.8 ± 12.5	0.87
BMI (kg/m^2^)		23.6 ± 4.6	32.1 ± 4.0	0.58
Patients with smoking history		24	32	0.48
Smoking history (pack per day × year)		28.1.7 ± 26.0	22.4 ± 17.9	0.27
O2 Saturation		96.8 ± 2.0	96.6 ± 2.1	0.65
FVC (%)		80.4 ± 13.8	77.3 ± 18.4	0.41
FEV1 (%)		66.8 ± 11.2	66.3 ± 20.3	0.86
FEV1/FVC (%)		61.0 ± 14.2	62.3 ± 16.2	0.72
DLCO (absolute value)		13.7 ± 3.7	14.9 ± 4.2	0.45
Acute exacerbations	Moderate	1	2	0.39
	Severe	4	7	0.27
Baseline	6MWD	471.0 ± 62.8	501.0 ± 73.3	0.07
	mMRC	1.2 ± 0.5	1.4 ± 0.7	0.57
	CAT	18.2 ± 7.0	17.5 ± 6.7	0.64
	SGRQ	29.6 ± 13.6	28.2 ± 13.3	0.64
	HADS	9.0 ± 5.7	10.0 ± 5.8	0.49

Data are expressed as mean ± SD, ratio, or number. PR, pulmonary rehabilitation; DTx, digital therapeutics; CG, control group; BMI, body mass index; FVC, forced vital capacity; FEV1, forced expiratory volume in one second; DLCO, diffusing capacity of the lungs for carbon monoxide; 6MWD, 6 min walk distance; mMRC, modified Medical Research Council; CAT, COPD assessment test; SGRQ, St. George’s respiratory questionnaire; HADS, hospital anxiety and depression scale.

**Table 2:** Comparison of participants exceeding and not exceeding 500 m in the 6 min walk test at baseline.

Characteristics		≥500 m Group (n = 34)	<500 m Group (n = 43)	*p*-Value
Group (DTx/CG ratio)		17:17	22:21	0.92
Age (years)		63.6 ± 10.4	67.2 ± 7.6	0.09
Sex, male/female ratio		26:8	38:5	0.17
Height (m)		1.66 ± 0.0	1.66 ± 0.1	0.74
Weight (kg)		63.4 ± 9.4	65.6 ± 16.0	0.48
BMI (kg/m^2^)		22.8 ± 2.9	23.7 ± 5.1	0.39
Patients with smoking history		22	34	0.05
Smoking history (pack per day × year)		21.0 ± 19.0	28.0 ± 23.3	0.17
O2 Saturation		97.6 ± 1.9	95.9 ± 1.9	0.00
FVC (%)		80.1 ± 15.4	77.4 ± 17.7	0.49
FEV1 (%)		72.5 ± 12.9	61.7 ± 18.6	0.00
FEV1/FVC (%)		68.2 ± 13.4	56.8 ± 15.0	0.00
DLCO (absolute value)		14.2 ± 3.8	14.1 ± 4.3	0.94
Acute exacerbations	Moderate	0	3	0.11
	Severe	4	7	0.50
Baseline	6MWD	547.2 ± 44.0	442.5 ± 49.9	0.00
	mMRC	1.1± 0.4	1.3 ± 0.8	0.25
	CAT	18.2 ± 4.9	17.4 ± 8.0	0.56
	SGRQ	24.6 ± 9.1	32.1 ± 15.2	0.01
	HADS	9.5 ± 6.2	9.7 ± 5.5	0.88
8 week follow up	6MWD	582.8 ± 58.1	489.9 ± 87.7	0.00
	mMRC	0.9 ± 0.4	1.3 ± 0.8	0.01
	CAT	14.4 ± 7.3	16.7 ± 8.5	0.22
	SGRQ	22.7 ± 10.0	33.3 ± 15.8	0.00
	HADS	8.5 ± 6.5	8.1 ± 6.0	0.77

Data are expressed as mean ± SD, ratio or number. PR, pulmonary rehabilitation; DTx, digital therapeutics; CG, control group; BMI, body mass index; FVC, forced vital capacity; FEV1, forced expiratory volume in one second; DLCO, diffusing capacity of the lungs for carbon monoxide; 6MWD, 6 min walk distance; mMRC, modified Medical Research Council; CAT, COPD assessment test; SGRQ, St. George’s respiratory questionnaire; HADS, hospital anxiety and depression scale.

**Table 3:** Impact of Digital Therapeutics and Baseline 500 m 6MWD Threshold on Distance Improvement in Home-Based Pulmonary Rehabilitation Participants.

Six MWD Results	DTx (n = 39)	CG (n = 38)	*p*-Value	≥500 m (n = 34)	<500 m (n = 43)	*p*-Value
Exceeded MCID	21	10	0.01	10	21	0.08
Gradual improvement	19	8	0.01	9	18	0.16
Gradual improvement over MCID	16	7	0.03	6	17	0.03
Gradual decline	0	3	0.08	0	3	0.08
Deterioration from the baseline	12	19	0.09	16	15	0.28

6MWD, 6 min walk distance; PR, pulmonary rehabilitation; DTx, digital therapeutics; CG, control group; MCID, minimal clinically important difference.

**Table 4:** Multivariate logistic regression analysis of possible predictive factors for favorable response in dyspnea scale and HRQOL after PR.

Independent Variables	mMRC Improvement	ΔCAT Exceeding MCID	ΔSGRQ Exceeding MCID
Groups (DTx vs. CG)	22.7, 1.8–287.2 ^(a)^	11.1, 2.7–46.6 ^(b)^	9.4, 2.1–43.2 ^(c)^
Age	0.9, 0.8–1.0	0.9, 0.8–1.0	1.0, 0.9–1.0
Male sex	0.6, 0.1–3.4	0.8, 0.2–4.2	1.1, 0.3–4.8
DLCO (absolute value)	0.5, 0.3–0.9 ^(a)^	0.9, 0.8–1.1	1.0, 0.9–1.2
Current smoker	0.5, 0.0–9.7	1.4, 0.2–7.9	0.3, 0.0–2.8
Comorbid liver dz.	0.0, -	0.6, 0.0–13.6	3.0, 0.1–62.6
Comorbid cancer	6.7, 0.0–12,074.7	2.1, 0.1–31.0	0.0, -

Data are expressed as odds ratio, 95% confidence interval. mMRC, modified Medical Research Council; Δ, change between baseline and week 8; CAT, COPD assessment test; SGRQ, St. George’s respiratory questionnaire; DTx, digital therapeutics; CG, control group; DLCO, diffusing capacity of the lungs for carbon monoxide; dz., disease. Statistically significant (*p* < 0.05) values in the logistic regression: ^(a)^ *p* = 0.02; ^(b)^ *p* = 0.001; ^(c)^ *p* = 0.004.

**Table 5:** Pearson’s correlation coefficients among change in 6MWD and patient-reported outcome measures of dyspnea and health-related quality of life.

		CAT at Week 8	ΔCAT	SGRQ at Week 8	ΔSGRQ
Δ6MWD	Pearson’s Correlation Coefficient	−0.29	−0.35	−0.36	−0.81
	*p*-value	0.01	0.00	0.38	0.24
	N	77	77	77	77

CAT, COPD assessment test; Δ, change between baseline and week 8; SGRQ, St. George’s respiratory questionnaire; 6MWD, 6 min walk distance.

## Data Availability

The study data are available upon request from the corresponding author.
